# P-946. Healthcare Worker Burden, Cost and Environmental Savings of Vancomycin Stewardship in Pediatric Intensive Care

**DOI:** 10.1093/ofid/ofaf695.1149

**Published:** 2026-01-11

**Authors:** Preeti Jaggi, Christine Lockowitz, Jeffrey S Gerber, Humza Agha, Kai Inoki, Evan E Facer, Rebecca Same, Valerie Yuenger, Leila Hojat, Kathleen Chiotos

**Affiliations:** Emory University, Atlanta, GA; St. Louis Children’s Hospital, St. Louis, Missouri; Children's Hospital of Philadelphia, Philadelphia, PA; Washington University in St. Louis School of Medicine, St. Louis, Missouri; Children's Hospital of Philadelphia, Philadelphia, PA; St. Louis Children's Hospital, St. Louis, Missouri; Children's Hospital of Philadelphia, Philadelphia, PA; St. Louis Children's Hospital, St. Louis, Missouri; Emory University School of Medicine; Children's Hospital of Philadelphia, Philadelphia, PA

## Abstract

**Background:**

Overuse of vancomycin is common in the pediatric intensive care unit (PICU). As a secondary analysis of an ongoing multicenter study focused reducing unnecessary empiric vancomycin use in three tertiary care pediatric intensive care units (PICUs), our objective was to estimate the impact of reducing vancomycin overuse on healthcare worker time, solid waste, and financial costs.

**Methods:**

We estimated vancomycin days saved by comparing the number of days of therapy in the preintervention (9/2022-8/2022) to the postintervention (11/2022-10/2023) periods across the three tertiary care PICUs. Pharmacy time to prepare a dose of vancomycin was estimated as the median time from completion of order verification to the time the dose is ready for delivery using DoseEdge pharmacy database timestamps. Order verification and drug delivery time were not included. Nursing administration time was calculated as the median time from drug delivery to completing the infusion, excluding infusion time, calculated over 10 direct observations at two sites. Average wholesale price, solid waste, and number of intravenous (IV) line accesses were estimated using a dose of 15 mg/kg/dose every 6 hours for a 33 kg child. Daily cost was estimated at $10.00/dose, per Lexicomp database, and solid waste, inclusive of preparation and drug administration, was estimated to be 0.37 kg/day, per published literature.

**Results:**

A total of 7,788 vancomycin DOT (98 DOT per 1000 patient days, PD) were administered during the pre-intervention period, versus 6,686 DOT in the post-intervention period (82 per 1000 PD), a reduction of 1,102 DOT (16 DOT per 1000 PD). Median pharmacists preparation time was 11:00 minutes (interquartile range [IQR] 6,17). Median nursing time to administer was 3:49 minutes (IQR 3:22, 7:16). The intervention avoided 4,408 (IV) accesses, 808 hours of pharmacy technician time, 294 hours of nursing time, 408 kg of solid waste, and $44,080 in drug cost. One day of vancomycin is associated with 4 intravenous accesses, $40, & 0.37 kg of waste and 1 hour of healthcare worker time.

**Conclusion:**

Reducing unnecessary vancomycin exposure can save significant healthcare worker time, cost and solid waste. Antibiotic stewards may consider highlighting additional benefits of antibiotic stewardship.

**Disclosures:**

Christine Lockowitz, PharmD, AbbVie: Grant/Research Support|Premier, Inc.: Honoraria Evan E. Facer, DO, AbbVie, Inc.: Grant/Research Support

